# Prostaglandin E_2_ affects in vitro maturation of bovine oocytes

**DOI:** 10.1186/s12958-020-00598-9

**Published:** 2020-05-11

**Authors:** Dorota Boruszewska, Ilona Kowalczyk-Zieba, Katarzyna Suwik, Joanna Staszkiewicz-Chodor, Joanna Jaworska, Krzysztof Lukaszuk, Izabela Woclawek-Potocka

**Affiliations:** 1 Department of Gamete and Embryo Biology, Institute of Animal Reproduction and Food Research, Polish Academy of Sciences, Tuwima 10 Str., 10-748 Olsztyn, Poland; 2 Department of Obstetrics and Gynecological Nursing, Faculty of Health Sciences, Medical University of Gdansk, M. Skłodowskiej-Curie 3a Str., 80-210 Gdansk, Poland; 3 INVICTA Fertility and Reproductive Center, Rajska 10 Str., 80-850 Gdansk, Poland

**Keywords:** Apoptosis, Cumulus cells, Embryo culture, Gene expression, In vitro fertilization (IVF), In vitro maturation (IVM), Mitochondria, Oocyte, Oxidative stress, Cow

## Abstract

The role of prostaglandin E_2_ (PGE_2_) in the successful resumption of oocyte meiosis and cumulus expansion has been well-documented. However, there remains very little information available on the influence of PGE_2_ on other processes that occur during oocyte maturation. In this study, we supplemented a maturation medium with PGE_2_ and monitored oocyte quality markers, glucose metabolism, mitochondrial status, oxidative stress, and apoptosis in the cumulus-oocyte complexes (COCs), using a well-established in vitro model of embryo production in cattle. We found that this increased availability of PGE_2_ during maturation led to an increase in the expression of genes associated with oocyte competence and improved the quality of blastocysts produced. Prostaglandin E_2_ also appeared to stimulate glucose uptake and lactate production in the COCs, both influencing the expression of enzymes involved in glycolysis and the hexosamine biosynthetic pathway. We found that PGE_2_ reduced intracellular reactive oxygen species levels, and simultaneously increased glutathione concentration and stimulated antioxidant gene expression in the oocyte. These results indicate that PGE_2_ has an important role in the protection of oocytes against oxidative stress. Mitochondrial membrane potential was also improved in PGE_2_-treated oocytes, and there was a reduction in the occurrence of apoptosis in the COCs. Promotion of an anti-apoptotic balance in transcription of genes involved in apoptosis was present in both oocytes and the cumulus cells. In summary, PGE_2_ could represent a novel autocrine/paracrine player in the mechanisms that can facilitate successful oocyte maturation and oocyte survival in the cow.

## Background

Prostaglandin E_2_ (PGE_2_) belongs to a group of cell signalling molecules that mediate many important reproductive processes, including ovulation, implantation, maintenance of luteal function and establishment of pregnancy [1, 2]. This important derivative of arachidonic acid is produced by prostaglandin endoperoxide synthase S1 (PTGS1) or PTGS2 (previously identified as cyclooxygenase enzymes COX1 and COX2) [3, 4], specific terminal synthases: two membrane bound enzymes (PTGES and PTGES2), and one cytosolic isoform (PTGES3 or cPGES) [5–7]. Once produced, PGE_2_ acts in both an autocrine or paracrine manner by binding to specific G protein-coupled receptors that have different but coinciding tissue distributions [8, 9]. Four isoforms of PGE_2_ receptors have been identified: PTGER1, PTGER2, PTGER3 and PTGER4 (commonly referred to as EP1, EP2, EP3, and EP4). These are known to activate different intracellular signalling pathways [8, 9] and can have a very influential role on the success of reproductive processes.

In mammals, PGE_2_ is the most abundant type of prostaglandin [10] and is the main prostaglandin secreted following PTGS2 induction in mice cumulus-oocyte complexes (COCs) [11]. There is a 6-fold increase in PTGS2 expression in competent COCs in human cumulus cells [12], with PTGS2 dependent PGE_2_ production also found in bovine COCs [13, 14]. The role of PGE_2_ during early embryogenesis has been studied in several species, including humans [15], rhesus monkeys [16], mice [17, 18], and various domestic animals [19–24]. Prostaglandin E_2_ is also involved in ovulation cascade events, including the expansion of cumulus cells and the expression of proteases associated with follicle rupture [15, 16, 18]**.** In these processes, PGE_2_ indirectly mediates LH action or directly acts on the expression of ovulatory genes, including *AREG*, *EREG*, *HAS2* and *TNFAIP6* [15, 18].

The influence of PGE_2_ on oocyte maturation and subsequent developmental competence has been the subject of extensive research, with authors typically assigning PGE_2_ an essential role in the successful resumption of oocyte meiosis and cumulus expansion [11, 17, 25–27]. However, little is known about the effect of PGE_2_ on other processes that occur during oocyte maturation. The present study was designed to investigate the effect of supplementing a maturation medium with PGE_2_ on several aspects of oocyte maturation, in a well-established in vitro model of maturation, fertilization, and embryo culture in cattle. We tested the hypothesis that during IVM, PGE_2_ effects the expression of oocyte quality markers, glucose metabolism, mitochondrial status, oxidative stress, and apoptosis in the COCs. Oocyte quality markers included their maturation level, embryonic development and blastocyst morphological quality. We measured the role of PGE_2_ on overall glucose metabolism and expression of genes involved in glucose metabolism in cumulus cells, as well as mRNA abundance of oocyte quality markers in cumulus cells. Mitochondrial status was determined by analysing mitochondrial distribution and mitochondrial membrane potential in the bovine oocytes. We also explored oxidative stress by assessing intracellular GSH and ROS levels and mRNA expression of antioxidation-associated genes in the oocytes. Finally, we evaluated the effect of PGE_2_ supplementation during IVM on apoptosis of COCs and the expression of genes involved in apoptotic pathway.

## Materials and methods

### Chemicals and suppliers

Culture media for the in vitro production of bovine embryos were procured from Minitube (Minitüb, Tiefenbach, Germany). All reagents and supplements for the in vitro culture were purchased from Merck (Sigma-Aldrich, Darmstadt, Germany) unless otherwise stated. Plastic dishes, four-well plates and tubes were acquired from Nunc (Thermo Fisher Scientific, Waltham, Massachusetts, USA). All the chemicals for reverse transcription were obtained from Invitrogen (Thermo Fisher Scientific, Waltham, Massachusetts, USA).

### Oocyte collection

Ovaries were collected from non-pregnant Holstein cows with normal cycles at a local slaughterhouse (Warmia, Biskupiec, Poland) and transported to the laboratory in sterile PBS at 32 °C. Cumulus-oocyte complexes (COCs) were acquired by aspiration from subordinate ovarian follicles that were less than 5 mm in diameter. Following assessment under a stereo microscope (Discovery V20, Carl Zeiss, Poznan, Poland; SZX7, Olympus, Warsaw, Poland), only COCs consisting of oocytes with homogeneous ooplasm without dark spots and surrounded by at least three layers of compact cumulus cells were selected for the study. COCs were washed twice in wash medium (M199; #M5017) supplemented with 20 mM HEPES (#H3784), 25 mM sodium bicarbonate (#S4019), 0.4% bovine serum albumin (BSA; #A9418) and 40 μg/ml gentamicin (#G1272). They were then washed in maturation medium.

### In vitro oocyte maturation

After washing, groups of 50 immature COCs were allowed to mature in four-well plates (#144444) containing 400 μl of maturation medium (TCM 199 Maturation Medium (19,990/0010)) supplemented with 0.02 IU/ml of pregnant mare’s serum gonadotropin (PMSG, #G4527), 0.01 IU/ml of human chorionic gonadotropin (hCG, #C0684) and 5% foetal bovine serum (FBS, #12106C). They were overlaid with 400 μl of mineral oil (#M5310) and incubated at 38.5 °C in a 5% CO_2_ humidified air atmosphere for 24 h for IVM. For the analysis of PGE_2_ effects on oocyte maturation, maturation medium was supplemented with 10 μM PGE_2_ (#14010; Cayman Chemical, Ann Arbor, Michigan, USA). The dose of PGE_2_ was based on previous bovine studies [26, 28]. Stock solution of PGE_2_ was dissolved in EtOH (#396420113; Avantor Performance Materials Poland, Gliwice, Poland) and stored at − 20 °C until use. On the day of the experiment, stock solution was further diluted by 1:100 in the maturation medium to reach the chosen concentration. The final concentration of EtOH (0.06%, v/v) in the medium was the same in all treatment groups. An additional group, containing the same volume of EtOH only, was included in each experiment as a control. Following maturation, a part of oocytes from each experimental group were completely denuded of cumulus cells by using hyaluronidase solution (H4272; Merck, Sigma-Aldrich, Darmstadt, Germany). These denuded oocytes were used to test the effect of PGE_2_ on oocyte maturation. To assess maturation ratio at the end of the maturation time, the denuded oocytes were observed at × 400 magnification under an inverted phase contrast microscope (CKX41, Olympus, Warsaw, Poland). Mature (MII) oocytes were characterized by extrusion of the first polar body without germinal vesicle in the cytoplasm, whereas the oocytes that had a germinal vesicle or had no germinal vesicle with non-extrusion of the first polar body were defined as immature. The maturation rates were calculated based on the total number of cultured oocytes. The results of developmental rates were derived from two independent experiments.

### In vitro embryo production

To assess embryo development of treated COCs, in vitro matured oocytes were fertilized. Pools of COCs from the control and PGE_2_-stimulated group were washed in fertilization medium (TL fertilization medium (19,990/0030) supplemented with 10 μg/ml of heparin (#08BK0110, WZF Polfa, Warsaw, Poland), 0.2 mM sodium pyruvate (#P3662) and 0.6% BSA). For IVF, frozen-thawed semen from the same bull was used throughout the experiment. After thawing, the semen was layered underneath capacitation medium (TL sperm capacitation medium (19,990/0020) supplemented with 0.1 mM sodium pyruvate, 0.6% BSA and 0.1 mg/ml gentamicin) and incubated for 1 h at 38.5 °C in a 5% CO_2_ and humidified air atmosphere to allow recovery of motile sperm using a swim-up procedure. After incubation, the upper two-thirds of the capacitation medium were recovered, centrifuged at 200×g for 10 min, the supernatant was removed, and the sperm pellet was diluted in an appropriate volume of fertilization medium to give a final concentration of 10^6^ motile sperm/ml. Groups of 50 COCs were co-incubated with spermatozoa in four-well dishes containing 400 µl of fertilization medium under 400 µl of mineral oil for 18 h at 38.5 °C, in a 5% CO_2_ humidified air atmosphere. The day of in vitro insemination was considered Day 0. At 18 h postinsemination (hpi) embryos were separated from cumulus cells by vortexing, washed three times in wash medium and at 48 hpi the cleavage rates were assessed. Embryos were then cultured in four-well dishes containing 400 µl of culture medium (SOF; synthetic oviduct fluid medium (19,990/0040) supplemented with amino acids: 10 µl/ml BME (#B6766) and 20 µl/ml MEM (#M7145)), 3.3 mM sodium pyruvate and 5% FBS under 400 µl of mineral oil. Culture was carried out at 38.5 °C in an atmosphere of 5% CO_2_, 5% O_2_, 95% N_2_ with high humidity for 7 days. The number and morphological quality of blastocysts were then determined. Embryo quality was evaluated based on the International Embryo Transfer Society (IETS) Manual [29]. The quality of the blastocysts were scored as follows: grade A = excellent and good; grade B = fair and moderate; grade C = poor; and grade D = dead or degenerating [29]. Embryos classified as quality grade A-C were counted for blastocyst formation and blastocyst quality ratios. The rates of development to the blastocyst stage were calculated based on the total number of fertilized oocytes. The results of developmental rates were derived from twelve independent experiments.

### RNA isolation and reverse transcription

For transcript level analysis, total RNA was extracted from oocytes and cumulus cells. Each experimental group used for total RNA isolation contained eight pools of 10 oocytes or all cumulus cells, separated from the respective oocytes. The oocytes and corresponding cumulus cells were suspended in the extraction buffer and were processed for RNA isolation according to the manufacturer’s instructions (#KIT0204; Arcturus PicoPure RNA Isolation Kit, Applied Biosystems, Thermo Fisher Scientific, Waltham, Massachusetts, USA). DNase treatment was performed for the removal of genomic DNA contamination using the RNase-free DNase Set (#79254, Qiagen, Hilden, Germany). Samples were stored at − 80 °C until reverse transcription. Reverse transcription (RT) was carried out using oligo (dT)12–18 primers (#18418–012) by Super Script III reverse transcriptase (#18080–044), in a total volume of 20 μl, to prime the RT reaction and produce cDNA. The RT reaction was carried out at 65 °C for 5 min and then 42 °C for 60 min, followed by a denaturation step at 70 °C for 15 min. RNase H (#18021–071) was used to degrade the RNA strand of an RNA-DNA hybrid (37 °C for 20 min). Reverse transcription products were diluted fifteen times and were stored at − 20 °C until real-time PCR amplification.

### Quantitative real-time PCR

Real-time PCR was conducted for the quantification of mRNA for the examined genes. The gene symbols, gene names, specific primer sequences and size of the amplified fragments of all transcripts are listed in Table 1. The results of mRNA abundance were normalized to the glyceraldehyde-3-phosphate dehydrogenase (*GAPDH*, an internal control) mRNA level and were expressed as arbitrary units. This housekeeping gene was chosen using NormFinder software, comparing three candidate genes: *GAPDH*, *β-actin* and *H2A.1* [30]. The primers were designed using an online software package (http://bioinfo.ut.ee/primer3/). Real-time PCR was performed using an ABI Prism 7900 (Applied Biosystems, Thermo Fischer Scientific, Waltham, Massachusetts, USA) sequence detection system and Maxima® SYBR Green/ROX qPCR Master Mix (#K0222, Thermo Fischer Scientific, Waltham, Massachusetts, USA). The PCR samples were analyzed in 384-well plates. Each reaction well (10 μl) contained 3 μl of RT product, 5 μM each of forward and reverse primers and 5 μl SYBR Green PCR master mix. In each reaction, we used a quantity of cDNA equivalent to 0.2 oocyte or cumulus cells from each COC. Real-time PCR was performed under the following conditions: 95 °C for 10 min, followed by 40 cycles of 94 °C for 15 s and 60 °C for 60 s. Subsequently, in each case PCR melting curves were obtained to ensure single-product amplification. To exclude the possibility of genomic DNA contamination in the RNA samples, the reactions were also performed either with blank-only buffer samples or in the absence of the reverse transcriptase enzyme. The specificity of the PCR products for all the examined genes was confirmed by gel electrophoresis and sequencing. The efficiency range for the target and internal control amplifications was between 95 and 100%. For the relative quantification of the mRNA levels, the real-time PCR Miner algorithm was used [31].

**Table 1:** Primers used for Real-time PCR

Gene Symbol	Gene Name	Primer sequence (5′-3′)	Fragment size, bp	Gen Bank accession no.
*PTGS2*	prostaglandin endoperoxide synthase 2	TGGGTGTGAAAGGGAGGAAA AAGTGCTGGGCAAAGAATGC	127	NM_174445.2
*PTGES*	microsomal prostaglandin E synthase 1	TGCTGGTCATCAAAATGTACG GCAGTTTCCCCAGGTATGC	300	NM_174443.2
*PTGES2*	microsomal prostaglandin E synthase 2	CCTCCTACAGAAAGGTGCC GTGATGATGTCTGCCAGGG	133	NM_001166554.1
*PTGES3*	cytosolic prostaglandin E synthase	TGCAAAGTGGTACGATCGG TAACCTTGGCCATGACTGG	253	NM 001007806
*PTGER1*	prostaglandin E_2_ receptor 1	GAGTCCCTTGCTGGTGGTGGT CTGGTTCCACGAGGCGAGGC	103	NM_001192148.1
*PTGER2*	prostaglandin E_2_ receptor 2	GCTCCTTGCCTTTCACGATTTT CTCAGGATGGCAAAGACCCAA	130	NM_174588.2
*PTGER3*	prostaglandin E_2_ receptor 3	CATACTGGGGCTCTCGGCG TCATTATCAGCAACGGCGACCA	200	NM_181032.1
*PTGER4*	prostaglandin E_2_ receptor 4	CATCCCGCTTGTGGTGCGAG AGGGTTTTTGCCGATGACTGG	76	NM_174589.2
*CTSB*	cathepsin B	GGCTCACCCTCTCCAGTCCT TCACAACCGCCTTGTCTGAA	136	NM 174031.2
*CTSK*	cathepsin K	GAACCACTTGGGGGACATGA GGGAACGAGAAGCGGGTACT	77	NM 001034435.1
*CTSS*	cathepsin S	CCGCCGTCAGCATTCTTAGT CATGTGCCATTGCAGAGGAG	99	NM 001033615.1
*CTSZ*	cathepsin Z	GGGGAGGGAGAAGATGATGG CCACGGAGACGATGTGGTTT	146	NM 001077835.1
*GLUT1*	glucose transporter 1	GATCCACAGAGCGCAGCC TGTCAGCTTCTTGCTGGTGG	90	NM_174602.2
*GLUT4*	glucose transporter 4	ATTGTGGCCATCTTTGGCTTCGTG AACCCATGCCGATGATGAAGTTGC	160	NM_174604.1
*GFPT1*	glutamine-fructose-6-phosphate transaminase 1	AAACACAGTCGGCAGTTCCA TGGCTACACCAATCTCAGGC	80	NM_001109961.1
*GFPT2*	glutamine-fructose-6-phosphate transaminase 2	GAGATGTGCGGAATCTTTGCC ACCTGCTGAGTCATAGCCTCT	120	NM_001076883.1
*PFKP*	phosphofructokinase	TCAGAGAACCGTGCCTGGAAGAAA TGACCACAAGCTCCTTGATCTGCT	112	NM_001193220.1
*LDHA*	lactate dehydrogenase A	TCTGGATTCAGCTCGCTTCCGTTA TTCTTCAGGGAGACACCAGCAACA	147	NM_174099.2
*CAT*	catalase	CTGGGACCCAACTATCTCCA GATGCTCGGGAGCACTAAAG	112	NM_001035386.2
*GPX4*	glutathione peroxidase 4	GTGCTCGCTCCATGCACGA CCTGGCTCCTGCCTCCCA	120	NM_174770.4
*FAS*	Fas cell surface death receptor	AAAAACTGGGGCTGCCCTTA CTTTGTGGGGGATGGAACAA	148	NM_174662.2
*FASLG*	Fas ligand	ACTACCGCCACCACCTCTGA GGCCACCAGAACCATGAAAA	85	NM_001098859.1
*TNFα*	tumor necrosis factor alpha	CCGCATTGCAGTCTCCTACC TGGGTTCATACCAGGGCTTG	110	NM_173966.2
*TNFR1*	tumor necrosis factor receptor 1	CCACTGGTGCTTCCAGCTCT TTTCCTTGGGGACAGGGACT	110	NM_174674.2
*TNFR2*	tumor necrosis factor receptor 2	CCCCAGGACTCTGGCTCTTT CCAAGACAGGACCCATCAGG	113	NM_001040490.1
*BAX*	BCL2 associated X, apoptosis regulator	GTGCCCGAGTTGATCAGGAC CCATGTGGGTGTCCCAAAGT	126	NM_173894.1
*BCL2*	BCL2 apoptosis regulator	GAGTTCGGAGGGGTCATGTG GCCTTCAGAGACAGCCAGGA	203	NM_001166486.1
*CASP8*	caspase 8	CTGAGAGAAGAGGCCCGTGA CCCGGCTTAGGAACTTGAGG	173	NM_001045970.2
*CASP3*	caspase 3	TGGTGCTGAGGATGACATGG GAGCCTGTGAGCGTGCTTTT	163	NM_001077840.1
*GAPDH*	glyceraldehyde-3-phosphate dehydrogenase	CACCCTCAAGATTGTCAGCA GGTCATAAGTCCCTCCACGA	103	NM_001034034.2

### PGE_2_ measurement

The concentration of PGE_2_ in maturation medium from the control group was established using the PGE_2_ ELISA kit (#ADI-901-001; Enzo Life Sciences Farmingdale, New York, USA) according to the manufacturer’s instructions. The standard curve for PGE_2_ ranged from 19.6 to 2500 pg/ml. The average intra- and inter-assay coefficients of variation were 10.7 and 12.8% respectively. The samples of six independent repeats were measured in duplicate. To determine PGE_2_ production, the measured concentration in media blanks was subtracted from the concentration in the control. Prostaglandin E_2_ production was expressed as pg/ml per COC.

### Glucose metabolism

After 24 h of IVM, the maturation medium from the control and PGE_2_-treated group was recovered and stored at − 20 °C until measurements were taken of glucose and lactate concentrations. These levels were determined using an ABL 800 FLEX analyzer (Radiometer Medical, Copenhagen, Denmark). To determine glucose uptake, the measured glucose concentration was subtracted from the concentration of glucose in media blanks (medium cultured without COCs). To determine lactate production the measured concentration in media blanks was subtracted from the concentration of the studied factors in experimental media. The results of glucose metabolism were derived from six independent experiments. Glucose uptake and lactate production were expressed as mg/dl per COC and mmol/L per COC, respectively.

### Mitochondrial staining

Mitochondrial distribution patterns were examined using MitoTracker™ Red CMXRos (#M7512; Invitrogen/Molecular Probes, Invitrogen (Thermo Fisher Scientific, Waltham, Massachusetts, USA). After 24 h of IVM, denuded oocytes were incubated with 0.1 μM MitoTracker^R^ probe for 20 min at 37 °C in the dark. The oocytes were then washed three times in 0.1% PVA in PBS and placed at 4% paraformaldehyde in PBS for 15 min at 37 °C. After fixation, the oocytes were washed twice in PBS and observed under an LSM 800 Confocal Laser Scanning Microscope System (Carl Zeiss, Poznan, Poland), using appropriate fluorescence filters with excitation/emission at 579 nm/599 nm. The oocytes were categorized into one of three groups based on the pattern of mitochondrial distribution: (1) oocytes with homogeneous distribution, where mitochondria were evenly distributed throughout the cytoplasm, (2) oocytes with semi-peripheral distribution, where mitochondria were unequally dispersed in the cytoplasm, and (3) oocytes with peripheral distribution, where mitochondria were located underneath the oolemma. This experiment was repeated three times with 25 COCs per treatment group. The mitochondrial distribution patterns were expressed as a percentage of total COCs.

Tetraethylbenzimidazolylcarbocyanine iodide dye (JC-1; JC-1 Mitochondrial Membrane Potential Assay Kit, #ab113850, Abcam, Cambridge, UK) was used to assess the mitochondrial membrane potential of the oocytes. After 24 h of IVM, denuded oocytes were washed three times in 0.1% PVA in PBS and incubated with 4 μM JC-1 solution for 30 min in humidified air with 5% CO_2_ at 38.5 °C in the dark. Oocytes were then washed twice in PBS and immediately observed under an LSM 800 Confocal Laser Scanning Microscope System (Carl Zeiss, Poznan, Poland) using an appropriate fluorescence filter: the JC-1 monomer (low mitochondria polarization/low membrane potential) was detected with a green filter (excitation: 495 nm, emission: 529 nm), and JC-1 aggregates (high mitochondria polarization/high membrane potential) were detected with a red filter (excitation: 565 nm, emission: 590 nm). The fluorescent images obtained were analyzed using ZEN blue 2.5 Pro Software (Carl Zeiss, Poznan, Poland) to determine the intensity of fluorescence in each oocyte. This experiment was repeated three times with 25 COCs per treatment group. Mitochondrial membrane potential was expressed as the ratio of red fluorescence intensity to green fluorescence intensity in each experimental group.

### Assessment of intracellular ROS and GSH levels

The intracellular ROS and GSH levels in the control and PGE_2_-treated oocytes were measured using 2′,7′-dichlorodihydrofluorescein diacetate (DCHFDA, #D6883; Merck, Sigma-Aldrich, Darmstadt, Germany) and ThiolTracker™ Violet Glutathione Detection Reagent (#T10095; Invitrogen/Molecular Probes, Thermo Fisher Scientific, Waltham, Massachusetts, USA), respectively. After 24 h of IVM, denuded oocytes were incubated with 100 μM DCHFDA or 20 μM ThiolTracker™ Violet dye solution for 30 min at 37 °C in the dark. The oocytes were then washed three times in 0.1% PVA in D-PBS and placed at 4% paraformaldehyde in D-PBS for 30 min at room temperature (RT). After fixation, the oocytes were washed twice in D-PBS and observed under an Axio Observer Microscope System (Carl Zeiss, Poznan, Poland) using appropriate fluorescence filters with excitation/emission at 495 nm/529 nm and 404 nm/526 nm for DCHFDA and ThiolTracker™ Violet, respectively. The fluorescent images obtained were analyzed using ZEN blue 2.5 Pro Software (Carl Zeiss, Poznan, Poland) to determine the intensity of fluorescence in each oocyte. The data were calculated as an average fluorescence intensity ratio. This experiment was repeated three times with 25 COCs per treatment group.

### Detection of apoptosis in COCs

Terminal-uridine nick-end labeling (TUNEL) was used to detect apoptotic cells in the COCs using an In Situ Cell Death Detection Kit, Fluorescein (#11684795910; Roche, Merck, Sigma-Aldrich, Darmstadt, Germany). At the end of the maturation time, COCs were fixed in 4% paraformaldehyde in PBS for 1 h at RT. The COCs were then permeabilized in 0.3% Triton X-100 (#T9284, Sigma-Aldrich, Germany) in 0.1% sodium citrate for 2 min on ice and washed twice in PBS. Before TUNEL labeling, positive control COCs were treated with 3000 U/ml DNase (#79254; Qiagen, Hilden, Germany) in the reaction buffer and incubated at RT for 10 min to induce DNA strand breaks. Following incubation, positive control and sample COCs were placed in 50 μl of TUNEL reaction mixture with the enzyme (terminal deoxynucleotidyl transferase) and incubated at 37 °C for 1 h in the dark. At the same time, negative control COCs were incubated in TUNEL label solution without the enzyme. Following incubation, COCs were washed three times in PBS, then stained with 10 μg/ml DAPI (#D9564; Merck, Sigma-Aldrich, Darmstadt, Germany) for 30 min at 30 °C in the dark. The COCs were observed under a confocal laser scanning microscope Olympus Fluoview FV10i (Olympus, Warsaw, Poland) using the DAPI filter to estimate the total number of nuclei and the FITC filter for assessment of TUNEL positive cells. The data was calculated as a percentage of FITC positive (apoptotic) cells within all detected DAPI positive cells. This experiment was repeated three times with 25 COCs per treatment group.

### Statistical analysis

Maturation rates, cleavage rates, rates of development to blastocyst and blastocyst quality were analyzed by Fisher’s exact test. The differences in the transcript levels of PGE_2_ synthases and receptors were determined by one-way ANOVA followed by Tukey’s multiple comparison test. The effects of treatment with PGE_2_ on mRNA expressions, apoptosis, mitochondrial membrane potential, and glucose, lactate, GSH and ROS levels were examined by Student’s t-test for independent pairs. Two-way ANOVA followed by the Tukey multiple comparison test was used to determine differences in mitochondrial distribution. All analyses were performed using the statistical software GraphPad PRISM 8.0 (GraphPad Software, La Jolla, California, USA), and the results are presented as the means ± SEM. Differences were considered statistically significant at the 95% confidence level (*P* < 0.05).

## Results

### Maturation rate, embryonic development and blastocyst morphological quality

The maturation rate of oocytes matured in the presence of PGE_2_ was not significantly different than those of control oocytes (61.36% vs. 74.51%, respectively; *P* = 0.061; Table 2). The cleavage rates assessed at 48 hpi were also similar in the control and PGE_2_-treated groups (81.55% vs. 82.35%, respectively; *P* > 0.1; Table 3). We found no difference in the proportion of oocytes reaching the blastocyst stage on Day 7 in the control compared to the PGE_2_-stimulated groups (26.94% vs. 31.25%, respectively; *P* > 0.1; Table 3). The analysis of the distribution of blastocyst quality showed that there were significantly more blastocysts given a grade of A or B in the PGE2-treated groups compared to the control (95.29% vs. 83.56%, respectively; *P* = 0.0006; Table 3).

**Table 2:** The effect of PGE_2_ treatment of in vitro oocyte maturation medium on maturation of bovine oocytes

Treatment	Total oocytes, n	Immature oocyte, n	Mature (MII) oocyte, n	Maturation rate, %	*P* value of maturation rate
control	88	34	54	61.36	0.061
PGE_2_	102	26	76	74.51	

Proportion of mature oocytes relative to the total number of oocytes

*P* values determined by Fisher’s exact test

**Table 3:** The effect of PGE_2_ treatment of in vitro oocyte maturation medium on the bovine embryo development and blastocyst morphological quality

Treatment	Fertilized oocytes, n	Cleaved embryos, n (%)	Blastocyst on Day 7, n (%)	Qualities A and B, n (%)
control	542	442 (81.55)^a^	146 (26.94)^a^	122 (83.56)^a^
PGE_2_	544	448 (82.35)^a^	170 (31.25)^a^	162 (95.29)^b^
		*P* = 0.7527	*P* = 0.1246	*P* = 0.0006

Proportion of the cleaved embryos on Day 2 and blastocysts on Day 7 of embryo culture relative to the total number of fertilized oocytes

Proportion of qualities A and B of blastocysts relative to the total number of blastocysts

*P* values determined by Fisher’s exact test

^a,b^ Different within each column

### PGE_2_ synthesis and action in COCs

**Fig. 1:**
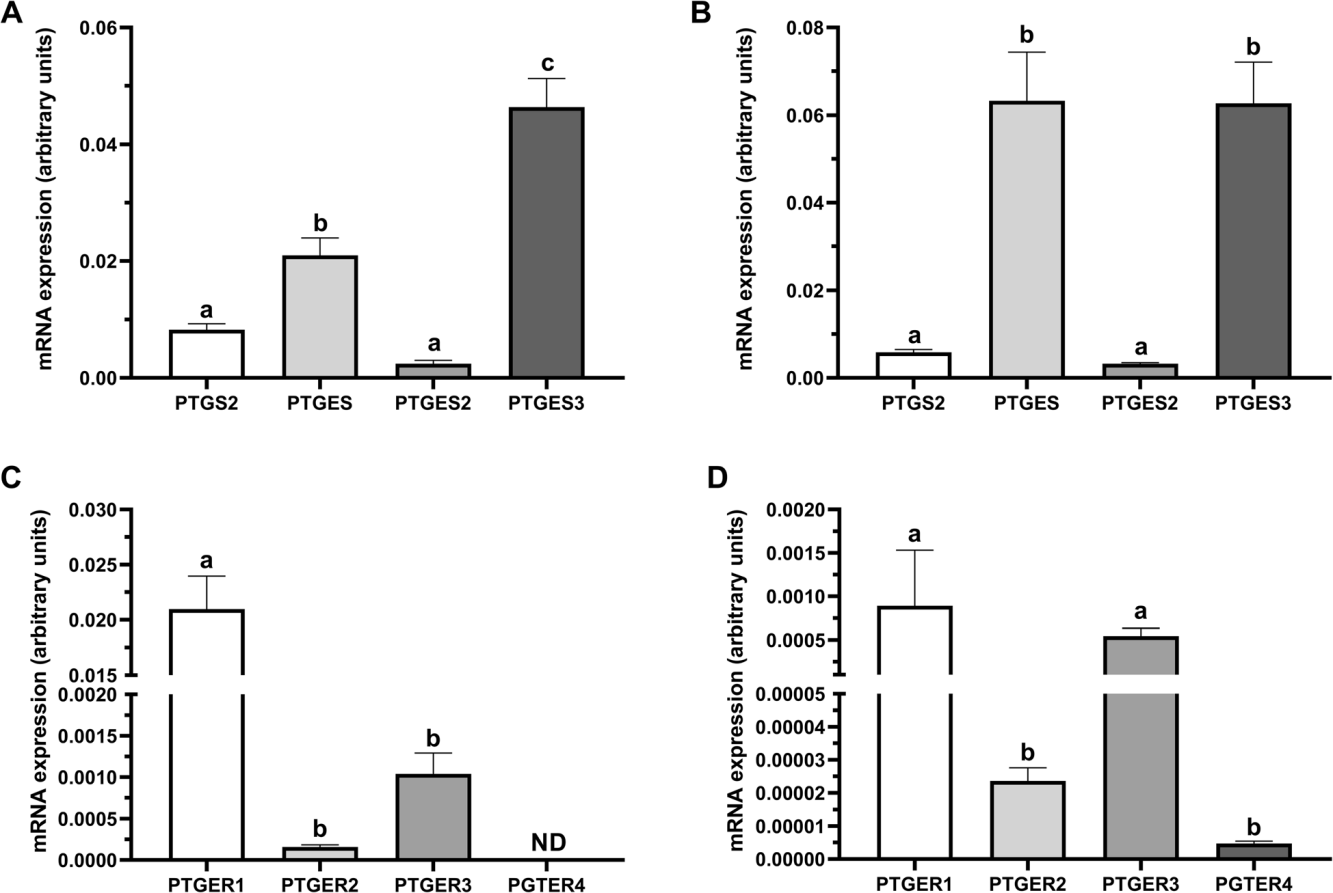
Transcription profiles of (**a**, **b**) PGE_2_ synthases: *PTGS2*, *PTGES*, *PTGES2*, *PTGES3*, and (**c**, **d**) PGE_2_ receptors: *PTGER1*, *PTGER2*, *PTGER3*, *PTGER4* in bovine oocytes (**a**, **c**) and cumulus cells (**b**, **d**) after IVM. The values are presented as arbitrary units and are expressed as the means ± SEM of eight independent repeats (10 oocytes and all cumulus cells separated from the respective oocytes in each replicate). Different letters indicate significant differences (*P* < 0.05), as determined by one-way ANOVA followed by Tukey’s multiple comparison test

We found the highest mRNA level of *PTGES3* in oocytes (*P* < 0.0001; Fig. 1a), and a significantly higher transcript abundance of *PTGES* compared to *PTGS2* and *PTGES2* (*P* < 0.05; Fig. 1a). Similarly, the transcript levels of *PTGES* and *PTGES3* were significantly higher than those of *PTGS2* and *PTGES2* in cumulus cells (*P* < 0.0001; Fig. 1b). A high mRNA level of *PTGER1* was also found in oocytes compared to *PTGER2* and *PTGER3* (*P* < 0.01; Fig. 1c). Transcripts for *PTGER4* were not identified in the oocytes (Fig. 1c). In turn, the mRNA abundance of *PTGER1* and *PTGER3* was significantly higher than those of *PTGER2* and *PTGER4* in the cumulus cells (*P* < 0.05; Fig. 1d). Prostaglandin E_2_ was also detected in the maturation medium after 24 h incubation of non-treated oocytes. The concentration of PGE_2_ in maturation medium amounts to 7.60 ± 3.88 pg/ml per COC.

### PGE_2_ effect on mRNA abundance of oocyte quality markers

**Fig. 2:**
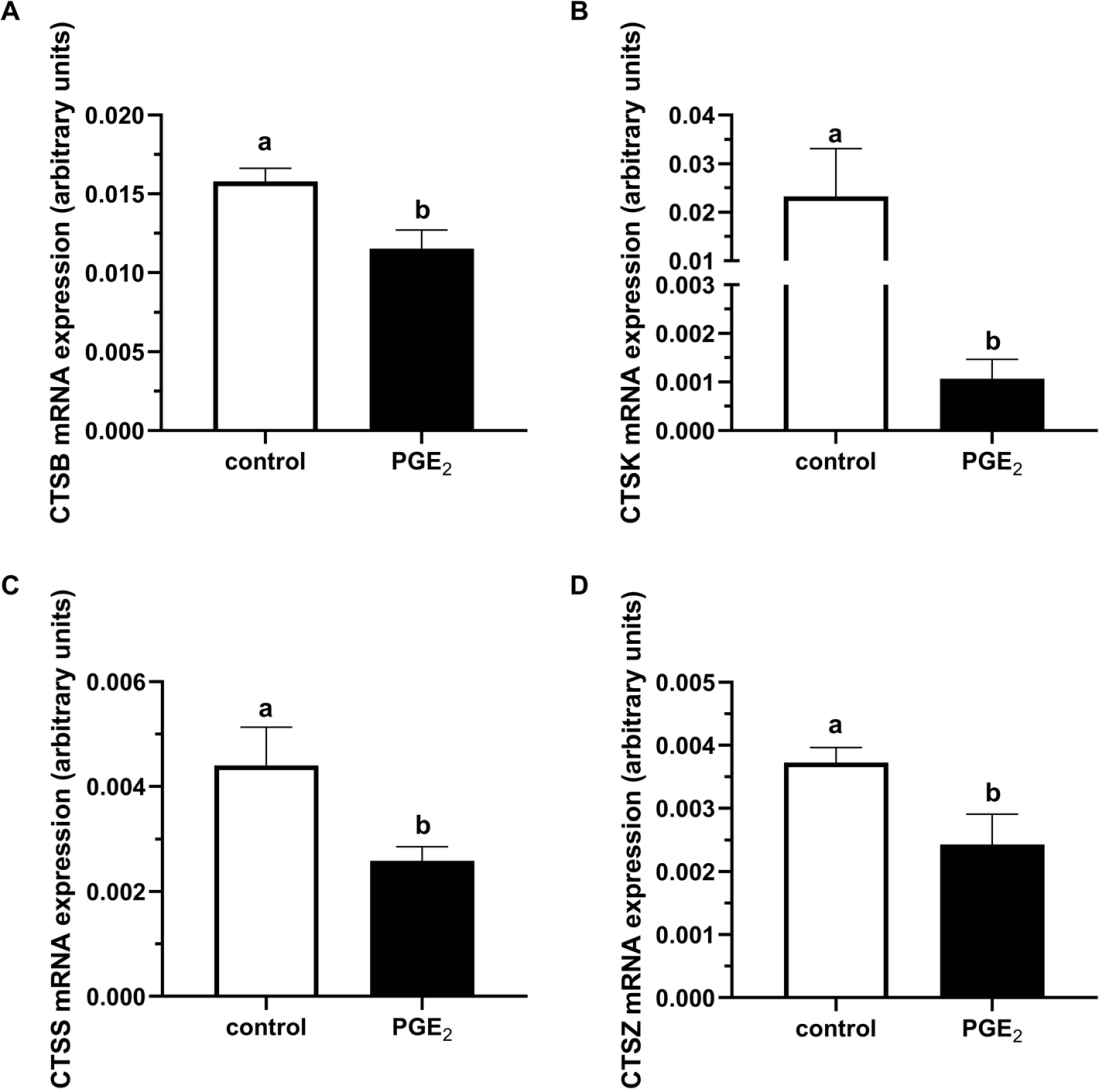
The effect of PGE_2_ (10 μM) treatment of maturation medium on mRNA abundance of cathepsins: **a***CTSB*, **b***CTSK*, **c***CTSS*, and **d***CTSZ* in cumulus cells. The values are presented as arbitrary units and expressed as mean ± SEM of eight independent repeats (all cumulus cells separated from 10 respective oocytes in each replicate). Different letters indicate significant differences (*P* < 0.05), as determined by Student’s t-test

Prostaglandin E_2_ supplementation of the maturation medium significantly decreased the transcript level of *CTSB*, *CTSK*, *CTSS*, and *CTSZ* in the cumulus cells (*P* < 0.05; Fig. 2a-d, respectively).

### PGE_2_ effect on glucose metabolism in the COCs

**Fig. 3:**
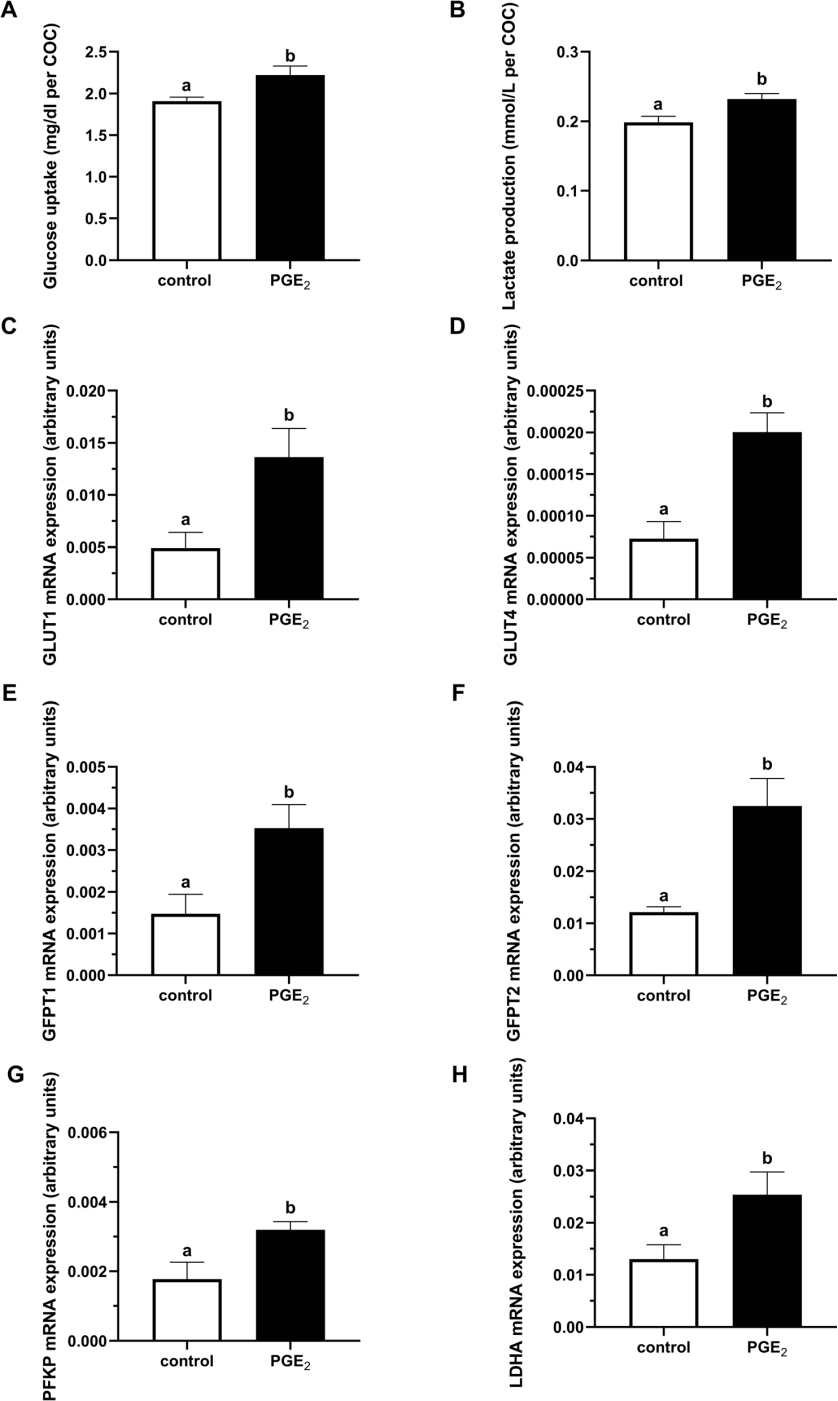
The effect of PGE_2_ (10 μM) treatment of oocyte maturation medium on (**a**) glucose uptake and (**b**) lactate production by COCs, and on mRNA abundance of genes involved in glucose metabolism: **c***GLUT1*, **d***GLUT4*, **e***GFPT1*, **f***GFPT2*, **g***PFKP*, and **h***LDHA* in cumulus cells. Glucose uptake and lactate production are expressed as mean ± SEM of six independent experiments and presented as mg/dl per COC and mmol/L per COC, respectively. The values of mRNA expression are presented as arbitrary units and expressed as mean ± SEM of eight independent repeats (all cumulus cells separated from 10 respective oocytes in each replicate). Different letters indicate significant differences (*P* < 0.05), as determined by Student’s t-test

During the IVM, PGE_2_ stimulated glucose uptake and lactate production by bovine COCs (*P* < 0.05; Fig. 3a, b, respectively). Moreover, in the cumulus cells, we found that addition of PGE_2_ to maturation medium significantly increased mRNA abundance of genes coding glucose transporters (*GLUT1* and *GLUT4*; *P* < 0.05; Fig. 3c, d, respectively), enzymes involved in hexosamine biosynthetic pathway (*GFPT1* and *GFPT2*; *P* < 0.05; Fig. 3e, f, respectively), and enzymes regulating glycolysis (*PFKP* and *LDHA*; *P* < 0.05; Fig. 3g, h, respectively).

### PGE_2_ effect on mitochondrial status in the oocytes

**Fig. 4:**
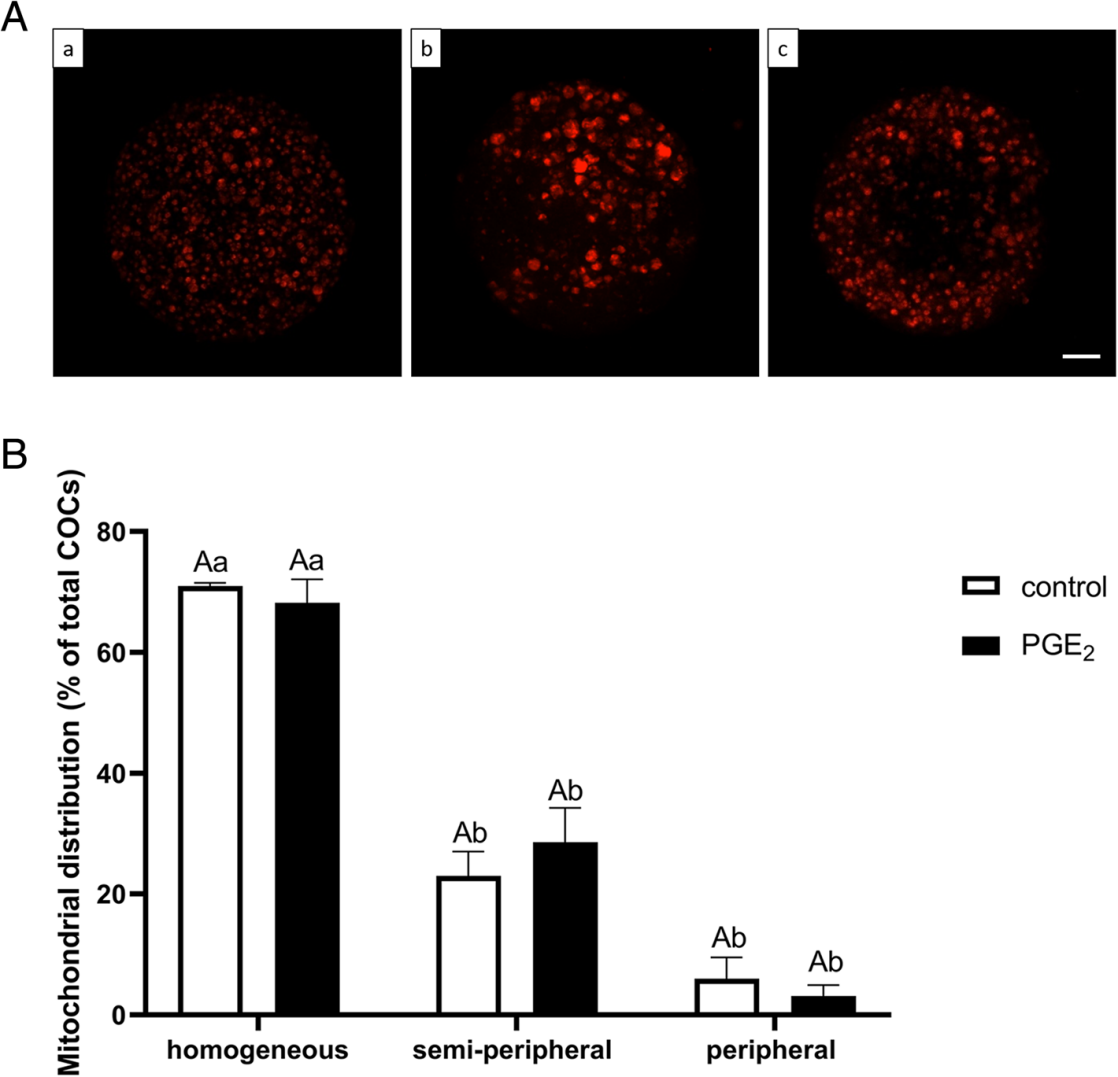
The effect of PGE_2_ (10 μM) on mitochondrial distribution pattern in bovine oocytes after IVM. Panel (A) depicts representative fluorescent images of mitochondrial (a) homogeneous, (b) semi-peripheral, and (c) peripheral distribution in bovine oocytes after IVM. Bars = 25 μm. Panel (B) depicts quantitative analysis of PGE_2_ effect on mitochondrial distribution pattern. The values are presented as percentage of COCs and expressed as mean ± SEM of three independent repeats (25 COCs per treatment group). Capital letters indicate statistical significance (*P* > 0.05) between two treatments whilst different small letters indicate significant differences (*P* < 0.05) within each treatment, as determined by two-way ANOVA followed by the Tukey multiple comparison test

**Fig. 5:**
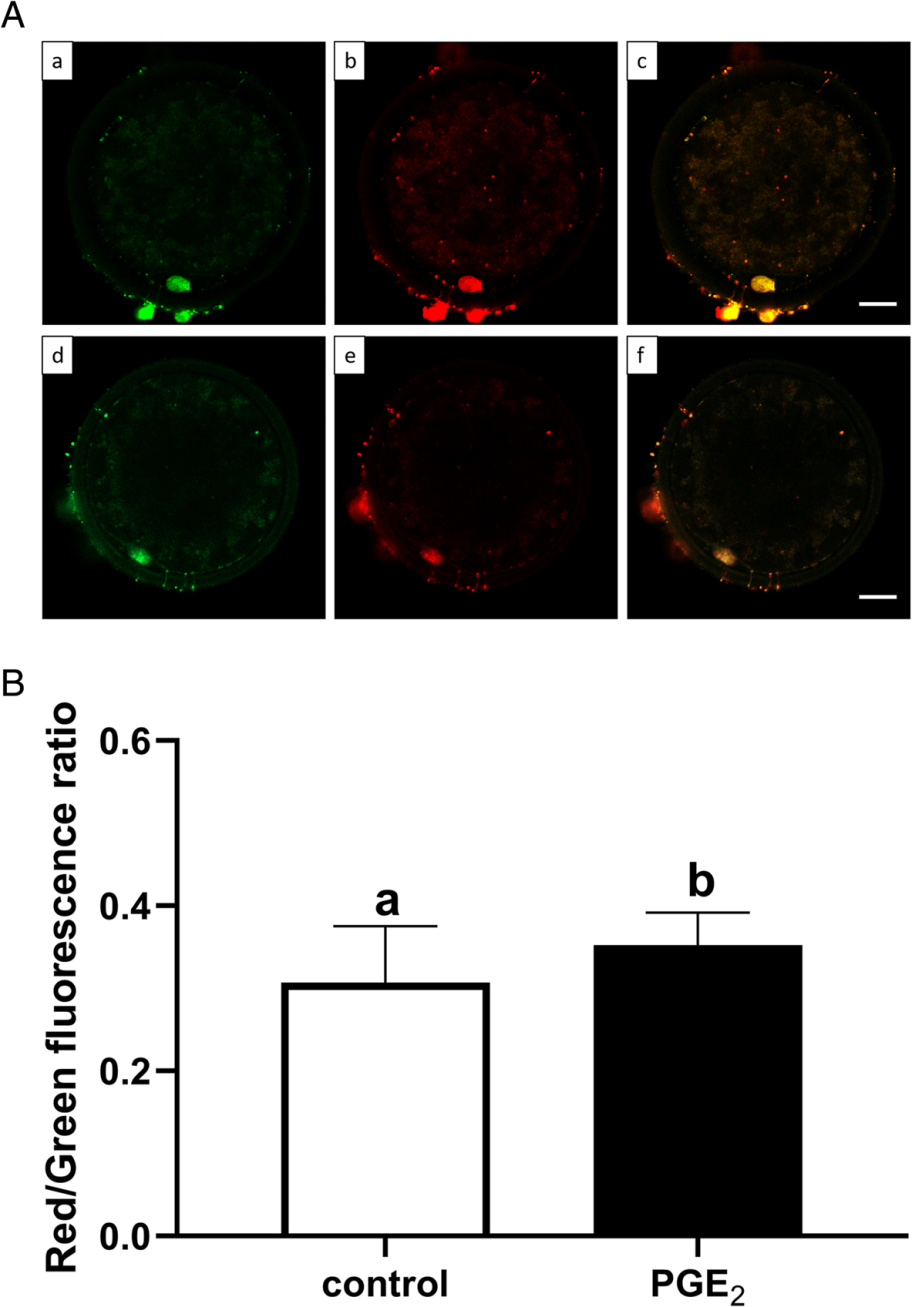
The effect of PGE_2_ (10 μM) on mitochondrial membrane potential (MMP) in bovine oocytes after IVM. Panel (A) depicts representative fluorescent images of oocytes with high (a-c) or low (d-f) polarization of mitochondria as detected by the JC-1 dye (a, d – green; b, e – red; c, f – merge fluorescence). Bars = 25 μm. Panel (B) depicts quantitative analysis of PGE_2_ effect on mitochondrial membrane potential. The values are presented as the ratio of red fluorescence intensity to green fluorescence intensity and expressed as mean ± SEM of three independent repeats (25 COCs per treatment group). Different letters indicate significant differences (*P* < 0.05), as determined by Student’s t-test

Figure 4A demonstrates representative fluorescent images of mitochondrial distribution in the bovine oocytes. In Fig. 5A, we present characteristic microphotographs of mitochondria with high and low polarization. The percentages of homogeneous, semi-peripheral and peripheral mitochondrial distribution were all similar in oocytes from the PGE_2_-treated group compared to the control oocytes (71.00% vs. 68.27, 23.00% vs. 28.6, 6.00% vs. 3.13%, respectively; *P* > 0.05; Fig. 4B). However, the ratio of red to green fluorescence intensity of JC-1 dye, which reflects the mitochondrial membrane potential of oocytes, was significantly increased in PGE_2_-treated oocytes compared with the control (*P* < 0.05; Fig. 5B).

### PGE_2_ effect on oxidative stress in the oocytes

**Fig. 6:**
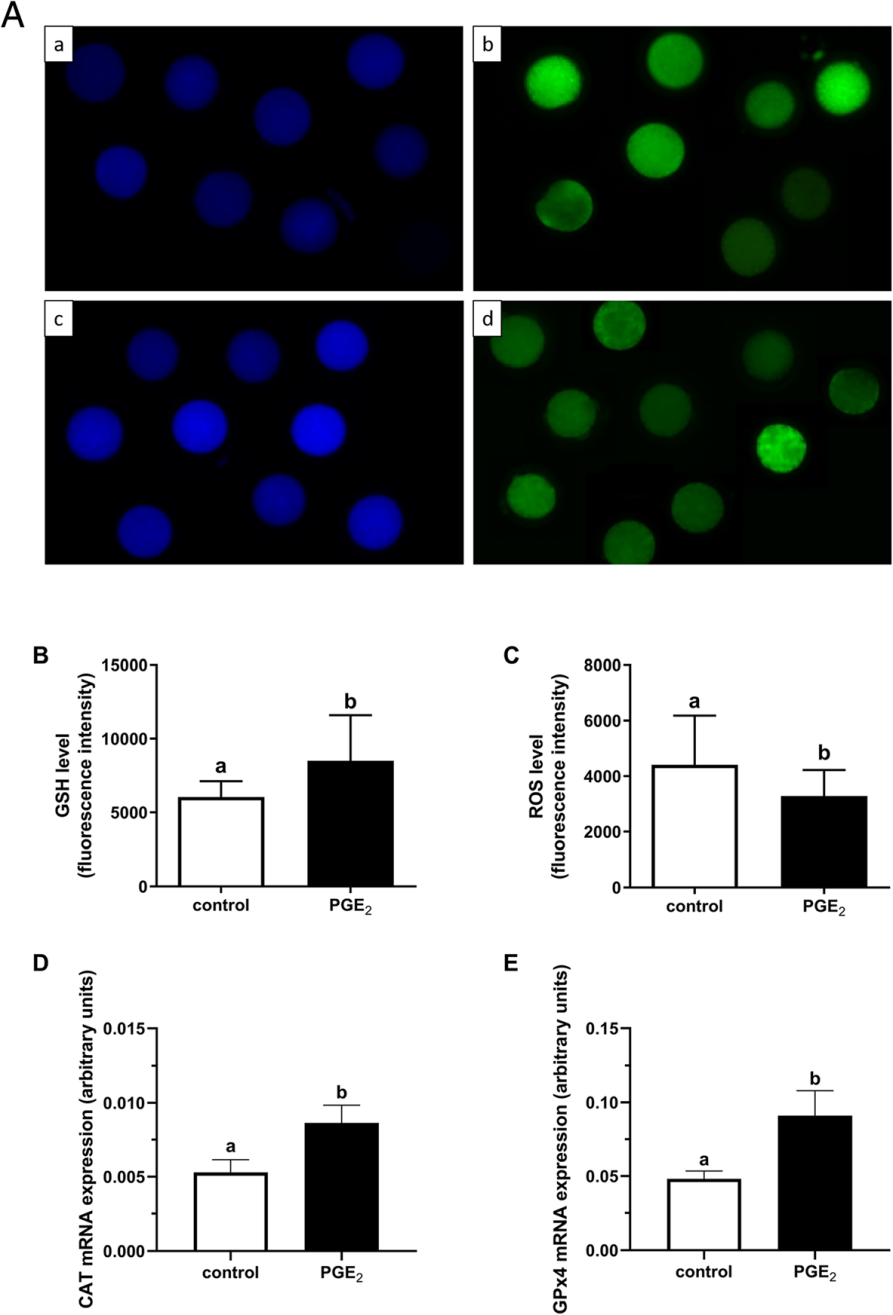
The effect of PGE_2_ (10 μM) on oxidative stress in bovine oocytes after IVM. Panel (A) depicts representative fluorescent images of intracellular GSH (a, c) and ROS (b, d) levels in the control (a, b) and PGE_2_-treated (c, d) oocytes. Bars = 50 μm. Panels (B) and (C) depict quantitative analysis of PGE_2_ effect on intracellular GSH and ROS levels, respectively. The values are presented as an average fluorescence intensity and expressed as mean ± SEM of three independent repeats (25 COCs per treatment group). Different letters indicate significant differences (*P* < 0.05), as determined by Student’s t-test. Panels (D) and (E) depict relative mRNA levels of the antioxidation-associated genes: *CAT* and *GPX4*, respectively, in the control and PGE_2_-treated oocytes. The values are presented as arbitrary units and expressed as mean ± SEM of eight independent repeats (10 oocytes in each replicate). Different letters indicate significant differences (*P* < 0.05), as determined by Student’s t-test

Figure 6A demonstrates representative fluorescent images of bovine oocytes, labelled for assessment of intracellular GSH and ROS levels, with GSH depicted by blue fluorescence and ROS by green fluorescence. Oocytes matured in the presence of PGE_2_ showed significantly higher intracellular level of GSH relative to control oocytes (*P* < 0.05; Fig. 6B). We also demonstrated a significantly greater decrease in intracellular ROS level in PGE_2_-stimulated oocytes compared to control oocytes (*P* < 0.05; Fig. 6C). Additionally, PGE_2_ treatment during IVM resulted in a significant increase in mRNA levels of antioxidation-associated genes in the oocyte (*CAT* and *GPX4*; *P* < 0.05; Fig. 6D, E, respectively).

### PGE_2_ effect on apoptosis in the COCs

**Fig. 7:**
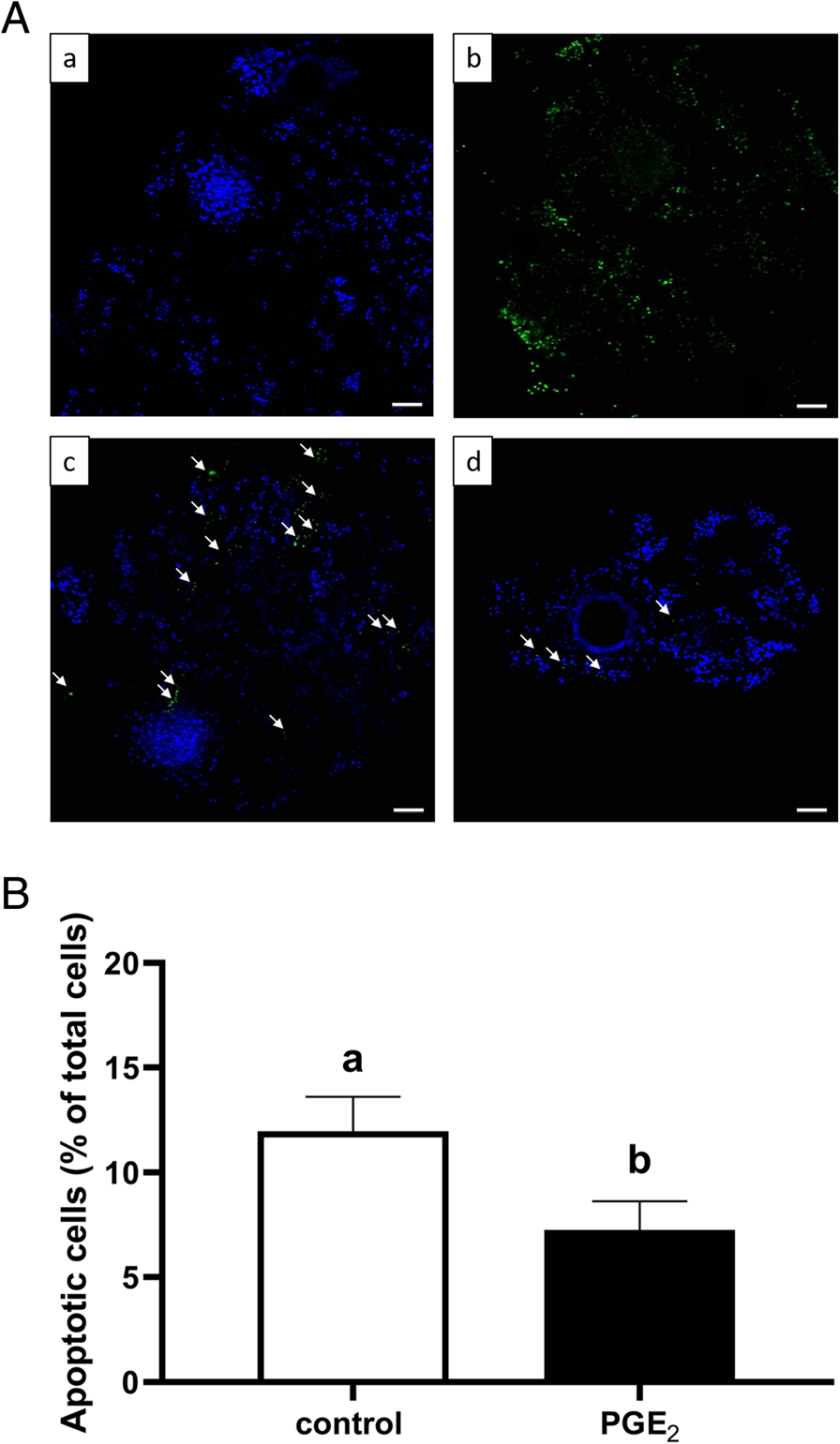
The effect of PGE_2_ (10 μM) treatment of oocyte maturation medium on apoptosis in COCs. Panel (A) depicts representative fluorescent images of bovine COCs used to TUNEL labeling: (a) negative control, (b) positive control, (c) control COCs, and (d) COCs matured in the presence of PGE_2_. White arrows indicate TUNEL stained apoptotic nuclei (green) in contrast to DAPI stained nuclei (blue). Bars = 100 μm. Panel (B) depicts quantitative analysis of PGE_2_ effect on apoptosis in COCs. The data are presented as a percentage of TUNEL positive apoptotic cells within all detected DAPI positive cells and expressed as mean ± SEM of three independent repeats (25 COCs per treatment group). Different letters indicate significant differences (*P* < 0.05), as determined by Student’s t-test

**Fig. 8:**
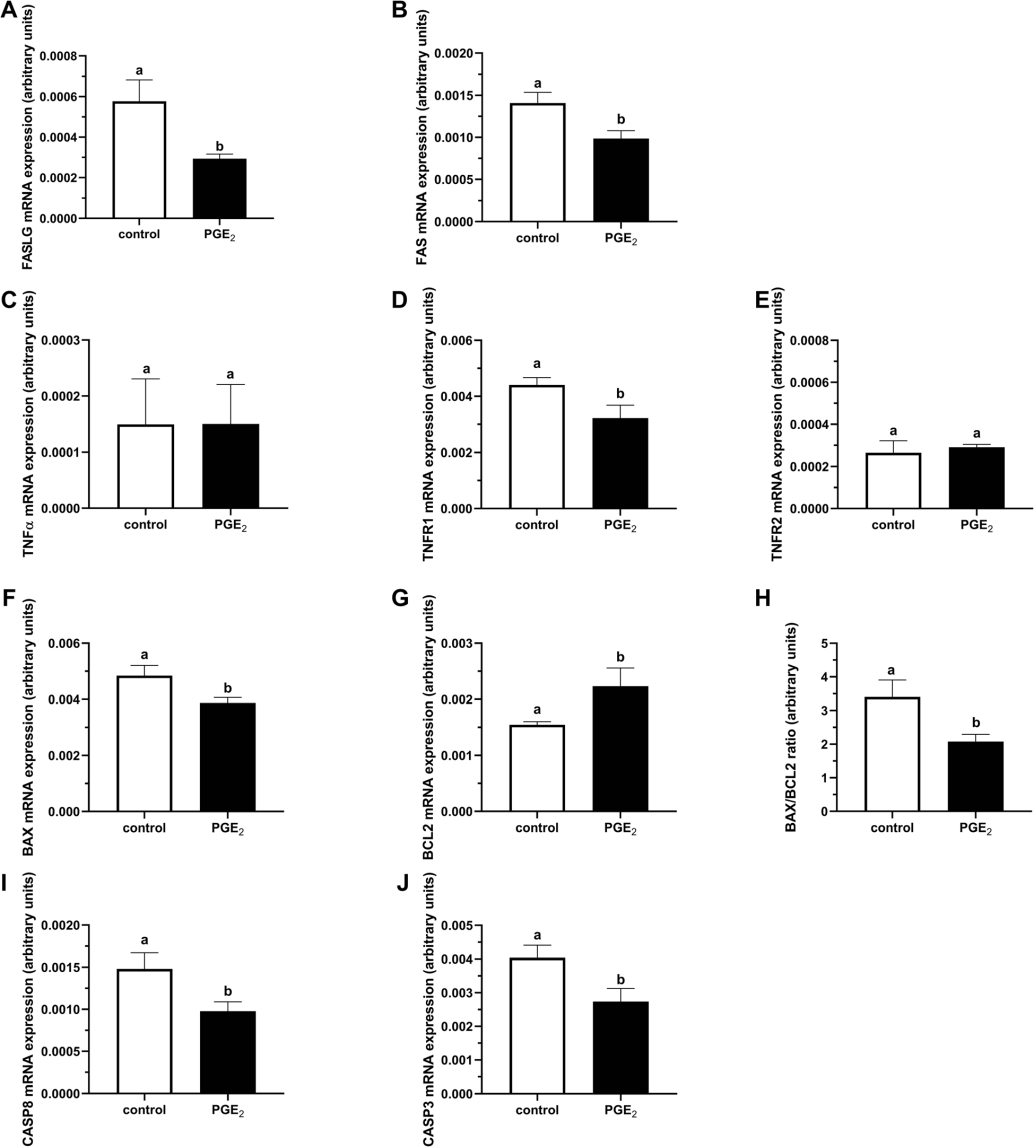
The effect of PGE_2_ (10 μM) treatment of maturation medium on mRNA abundance of factors involved in apoptosis: **a***FASLG*, **b***FAS*, **c***TNFα*, **d***TNFR1*, **e***TNFR2*, **f***BAX*, **g***BCL2*, **h***BAX/BCL2* ratio, **i***CASP8*, and **j***CASP3* in oocytes. The values are presented as arbitrary units and expressed as mean ± SEM of eight independent repeats (10 oocytes in each replicate). Different letters indicate significant differences (*P* < 0.05), as determined by Student’s t-test

**Fig. 9:**
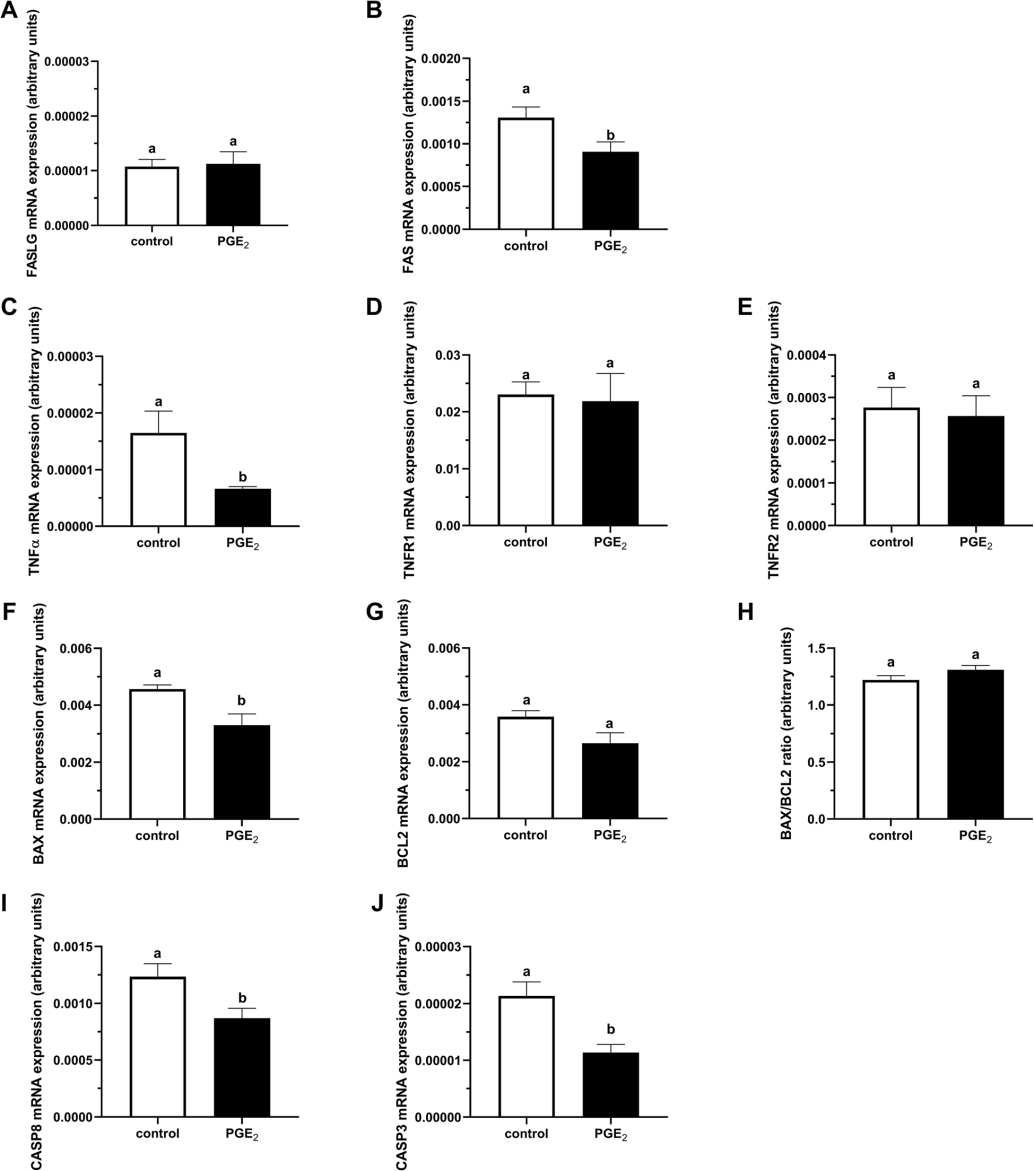
The effect of PGE_2_ (10 μM) treatment of maturation medium on mRNA abundance of factors involved in apoptosis: **a***FASLG*, **b***FAS*, **c***TNFα*, **d***TNFR1*, **e***TNFR2*, **f***BAX*, **g***BCL2*, **h***BAX/BCL2* ratio, **i***CASP8*, and **j***CASP3* in cumulus cells. The values are presented as arbitrary units and expressed as mean ± SEM of eight independent repeats (all cumulus cells separated from 10 oocytes in each replicate). Different letters indicate significant differences (*P* < 0.05), as determined by Student’s t-test

Figure 7A demonstrates representative fluorescent images of bovine COCs used for TUNEL labeling, with DNA fragmentation depicted by green fluorescence and total cells by blue fluorescence. The COCs matured in the presence of PGE_2_ showed a significantly reduced proportion of TUNEL positive apoptotic cumulus cells relative to control COCs (11.98% vs. 7.25%, respectively; *P* < 0.05; Fig. 7B). Moreover, in oocytes we found significantly lower mRNA abundance of genes coding for proteins linked to extrinsic apoptosis (*FASLG*, *FAS*, *TNFR1*; *P* < 0.05; Fig. 8a, b, d, respectively), intrinsic apoptosis (*BAX*; *P* < 0.05; Fig. 8f), and caspases (*CASP8* and *CASP3; P* < 0.05; Fig. 8i, j, respectively) in the PGE_2_-treated group compared to control. We also found a higher mRNA level of antiapoptotic *BCL2* and a lower BAX/BCL2 ratio in PGE_2_-stimulated oocytes compared to control oocytes (*P* < 0.05; Fig. 8g, h, respectively). In cumulus cells, we showed significantly lower mRNA abundance of genes coding for proteins associated with extrinsic apoptosis (*FAS*, *TNFα*; *P* < 0.05; Fig. 9b, c, respectively), mitochondrial apoptosis pathway (*BAX; P* < 0.05; Fig. 9f) and caspases (*CASP8* and *CASP3; P* < 0.05; Fig. 9i, j, respectively) in the PGE_2_-treated group compared to control.

## Discussion

### PGE_2_ synthesis in bovine COCs after IVM

This study details the mRNA expression of enzymes involved in PGE_2_ synthesis and the PGE_2_ receptors present in bovine oocytes and cumulus cells. These account for the ability of the bovine COCs to produce and secrete PGE_2_ during IVM. Bovine COCs therefore appear to be both a source and a target of PGE_2_ action, and PGE_2_ may be involved in cellular signaling between the oocyte and cumulus cells during maturation. In earlier studies of bovine COCs, transcript coding for *PTGS2* was detected in cumulus cells but not in the oocyte [13, 26]. However, Nuttinck et al. [14] and Marei et al. [27] examined the mRNA expression profile of PGE_2_ synthases in whole bovine COCs and found that expression of *PTGES* was significantly higher in mature than immature COCs. Moreover, the induction of *PTGES* expression coexisted with the induction of *PTGS2* expression and PGE_2_ secretion by maturing COCs, suggesting the involvement of a PTGS2/PTGES pathway during the maturation process [14]. We found the highest expression of *PTGES* and *PTGES3* in denuded oocytes and cumulus cells, which partially agrees with results obtained in whole COCs, where a significantly higher level of *PTGES* is found [14, 27]. Similar to earlier studies [27, 32], we showed that bovine COCs release PGE_2_ during IVM. In the expression of PGE_2_ receptors, only *PTGER2* mRNA has previously been found in bovine oocytes, while mRNA expression of *PTGER2*, *PTGER3* and *PTGER4* were detectable in cumulus cells [26]. For the whole bovine COCs, *PTGER2* and *PTGER3* were found to be expressed, while very low levels of *PTGER4* expression were detectable and *PTGER1* was not detected [33]. However, in this study we found the mRNA level of *PTGER1* to be the highest and no transcripts of *PGTER4* in oocytes. The highest expression of *PTGER1* and *PTGER3* was found in cumulus cells. The present data, concerning cell specific expression of PGE_2_ synthases and PGE_2_ receptors in bovine COCs, confirm the inherence of a PGE_2_ autocrine/paracrine regulatory pathway during oocyte maturation.

### PGE_2_ influence on oocyte developmental competence

In human cumulus cells, higher *PTGS2* expression is reported in competent COCs, from which oocytes develop into better quality embryos [12]. Addition of PGE_2_ to the fertilization medium also increases the cleavage rate as well as bovine oocytes with low cleavage rates produce less PGE_2_ than oocytes with high cleavage rates [32]. This suggests that PGE_2_ could be involved in the acquisition of oocyte developmental competence. We demonstrated that in bovine cumulus cells, PGE_2_ supplementation of maturation medium reduces the expression of *CTSB*, *CTSK*, *CTSS* and *CTSZ*. A higher expression of these enzymes is associated with developmental incompetence of bovine oocytes and thus this expression could serve as oocyte quality markers [34]. In turn, PGE_2_ added to the oocyte maturation medium had no effect on the number of matured oocytes, cleaved embryos or gained blastocysts. This supports previous studies that suggest the presence of PGE_2_ during IVM and IVF does not influence the proportion of cleaved embryos and blastocysts on Day 8 of culture [28]. Inhibition of PTGS2 during IVM, resulting in a reduced maturation rate at 22 h of IVM and decreased embryo output rates on Day 6 of culture, had no more effect on both rates at a further time point of IVM or on Day 7 of culture [26, 27]. Moreover, the addition of PGE_2_ to maturation medium with inhibited PTGS2 activity abrogated oocyte maturation and early embryonic developmental defects [26, 27]. The authors suggested that these adverse effects of PTGS2 inhibition during IVM are due to a delay in nuclear maturation and in the kinetics of early embryonic development [26, 27]. However, our study demonstrated that, although addition of PGE_2_ during IVM had no effect on the normal embryonic development rates, it enhanced the number of better-quality blastocysts. This suggests that PGE_2_ has a role in increasing the quality of the bovine oocytes.

### PGE_2_ action on glucose metabolism

The terminal differentiation of the COCs, leading to the resumption of meiosis and cumulus expansion, includes processes that require substantial energy from several substrates [35, 36]. Previous studies have suggested that glucose metabolism has a positive effect on oocyte maturation and subsequent blastocyst development [36]. Additionally, the dynamics of the glucose metabolism can be used as a predictive marker of oocyte quality [37]. The lower glucose metabolism in oocytes from pre-pubertal cows in comparison to adult cows [37], as well as the expression of genes coding for *GLUT1* in bovine blastocysts produced in vitro compared with in vivo obtained embryos [38], could account for its relevance of the developmental competence of bovine oocytes and embryos. In our study, addition of PGE_2_ to the oocyte maturation medium increased mRNA expression of *GLUT1* and *GLUT4*. Oocyte capacity for glucose uptake and metabolism is limited and thus cumulus cells provide the intermediates of glucose metabolism, including pyruvate or lactate, to the oocyte [39, 40]. We found that PGE_2_ increased glucose uptake and lactate production by bovine COCs and simultaneously stimulated expression of genes encoding glycolytic enzymes (*PFKP* and *LDHA*). During IVM of bovine oocytes, the linear correlation between glucose utilization and lactate production suggests that COCs preferentially consume glucose via glycolysis [41]. The presence of pyruvate or lactate plus NAD in the maturation medium enhances the maturity level of denuded oocytes, suggesting that the oocytes use them as substrates for energy production [39]. In the present study, we demonstrated that addition of PGE_2_ to the maturation medium stimulated the expression of genes encoding *GFPT1* and *GFPT2* in the cumulus cells. These are the rate-limiting enzymes in the hexosamine pathway, in which glucose is converted to glucosamine, a substrate for hyaluronic acid, a major component of the cumulus extracellular matrix [42–44]. It has been shown that a quarter of glucose used by bovine COCs in the later period of IVM is not metabolized through glycolysis [45]. However, addition of glucosamine to IVM medium leads to a decrease in glucose depletion and reduces the requirement of glucose for synthesis of matrix components [41], demonstrating a shift from glucose metabolism to the hexosamine pathway [40]. Therefore, the results of our study suggest that PGE_2_ increased glucose uptake and stimulated lactate production in bovine COCs, simultaneously affecting the expression of glycolytic enzymes. This indicates a role for PGE_2_ in directing glucose metabolism down the glycolytic pathway. In addition, PGE_2_ influence on the expression of enzymes involved in the hexosamine pathway, together with evidence of the role of PGE_2_ on cumulus expansion [27, 33], suggests it has a role in directing COCs glucose consumption towards the end of IVM to generate extracellular matrix.

### PGE_2_ action on oxidative stress and mitochondrial status

During ovarian follicle and oocyte development, ROS have important biological roles in the stimulation of meiotic resumption, extrusion of the first polar body, and in promoting the follicular wall rupture to allow the release of oocytes during ovulation [46–48]. However, high levels of ROS and low levels of antioxidants in follicular fluid in women are associated with a reduced pregnancy outcome after intracytoplasmic sperm injection [49, 50]. An increased level of ROS during IVM also causes disturbances in chromosomal distribution, alterations in microtubules on MII spindles, and aneuploidy in mice oocytes. This suggests that oxidative stress can induce chromosomal errors and effect oocyte developmental potential [51, 52]. A higher intrafollicular level of DNA damage biomarker was found in women with high rates of degenerated oocytes, suggesting again the association between oxidative stress and oocyte quality [53]. The authors also demonstrated a lower rate of matured mice oocytes after ROS treatment [53]. Moreover, bovine embryos cultured in atmospheric oxygen tension show higher ROS concentrations, lower blastocyst rates, lower total blastomere numbers, and differences in the expression of DNA-dependent transcription factors related to multiple pathways important for early embryo development [54]. This indicates that the oxidative stress may cause developmental failures and disturb embryo viability. In our study, we found that the addition of PGE_2_ to IVM medium reduced intracellular ROS level, simultaneously increasing GSH concentration and stimulating mRNA expression of antioxidant genes *CAT* and *GPX4* in the oocyte. One of the main roles of GSH is the maintenance of the redox status in cells, thereby protecting them from oxidative injuries [55, 56]. Optimal cumulus expansion during IVM of bovine oocytes is dependent on intracellular GSH content [57], while high GSH level during IVM improves the efficiency of embryo development [58]. Thus, our study suggests that PGE_2_ treatment during IVM of bovine oocytes ensures an adequate GSH accumulation for subsequent embryo development. Imbalance between ROS and antioxidants leads to irreversible damage of mitochondrial DNA and alteration of lipids and proteins of the inner membrane, resulting in mitochondrial dysfunction and finally in cell death [59]. In the present study, we found that the mitochondrial membrane potential of oocytes was improved by addition of PGE_2_ to the maturation medium. Since oxidative injury can induce the release of apoptogenic factors from the mitochondria, leading to the apoptosis of the cell [60], our data imply that PGE_2_ may protect bovine COCs from both oxidative stress and apoptosis during IVM.

### PGE_2_ action on apoptosis

Apoptosis of embryonic cells is a physiological process that occurs during embryo development and can be an indicator of embryo quality. The negative relationship between embryonic cell number and incidence of the TUNEL reaction is documented in both murine [61] and bovine [62, 63] embryos. This suggests that apoptosis may have an important role in appropriate embryo development, since blastocysts containing higher numbers of cells develop to form better quality embryos [64]. The degree of apoptosis in bovine cumulus cells is also negatively correlated with oocyte developmental capacity [65]. In cows, DNA damage detected by TUNEL labeling is also observed in morphologically fragmented oocytes and embryos [63, 66]. Results from the present study show that the presence of PGE_2_ during oocyte maturation reduced the number of TUNEL-positive apoptotic nuclei in bovine COCs. Similarly, supplementation of IVM and IVF medium with PGE_2_ reduces the apoptotic activity in blastocysts [28]. Apoptosis in blastocysts is also increased by PTGS2 inhibition during the IVM/IVF period, whereas addition of PGE_2_ overrides this effect, suggesting a role for PGE_2_ in the enhancement of embryonic cell survival during preimplantation development [28]. The incidence of apoptosis is regulated by numerous genes that are involved in either the extrinsic or intrinsic/mitochondrial pathways. Both apoptotic pathways stimulate the CASPs activity, leading to the execution of the apoptotic program [67, 68]. Thus, we examined through which pathway PGE_2_ could exert its prosurvival effect during IVM. The presence of PGE_2_ inhibits the expression of genes coding for proteins related to extrinsic apoptosis, including *FASLG*, *FAS*, *TNFR1* in oocytes and *FAS*, *TNFα* in cumulus. *CASP8* and *CASP3* are also affected in both oocytes and cumulus cells. In terms of the mitochondrial pathway, PGE_2_ increases antiapoptotic *BCL2* expression, decreases proapoptotic *BAX* expression, and reduces BAX/BCL2 ratio in oocytes. It also inhibits mRNA expression of *BAX* in cumulus cells. The treatment of IVM and IVF medium with PGE_2_ has been found to increase the ratio of BCL2/BAX mRNA expression in bovine embryos [28]. Furthermore, inhibition of PTGS2 expression during IVM and IVF decreases the BCL2/BAX ratio, whereas addition of PGE_2_ reverses this effect [28]. The BAX/BCL2 protein ratio has been found to be correlated with the developmental competence of bovine oocytes and embryos [66]. Other work found that mRNA expression of proapoptotic *BAX* and *CASP3* in bovine early stage embryos was associated with the DNA fragmentation detected in blastocysts, implying that expression of these genes may serve as a marker of embryo viability [63]. Thus, our study provided evidence that PGE_2_ during IVM is involved in the regulation of apoptotic activity, decreasing the extent of apoptosis in the COCs and altering the expression of apoptosis related genes. Moreover, results of this study, together with the previous data from Nuttinck et al. [28], suggest that periconceptional PGE_2_ supports antiapoptotic events during bovine early embryo development following IVM.

## Conclusions

This study provides evidence, from both functional and gene expression studies, that PGE_2_ autocrine/paracrine pathway is involved in the control of processes that occur during oocyte maturation in the cow. The exposure of COCs to PGE_2_ during IVM stimulated the expression patterns of genes associated with oocyte competence and enhanced the number of better-quality blastocysts. To the best of our knowledge, we are also the first to demonstrate that PGE_2_ has an influence on glucose metabolism. Furthermore, PGE_2_ appeared to improve the resistance of oocytes to oxidative attack and decreased the occurrence of apoptosis in the COCs, which may be reflected in oocyte viability. We suggest that PGE_2_ could represent an important player in the mechanisms that can facilitate successful oocyte maturation and oocyte survival in cattle.

## Data Availability

The datasets used and/or analysed during the current study are available from the corresponding author on reasonable request. All data generated or analysed during this study are included in this published article.

## Abbreviations

ANOVA: Analysis of variance
AREG: Amphiregulin
BAX: BCL2 associated X, apoptosis regulator
BCL2: BCL2 apoptosis regulator
BME: Basal medium Eagle amino acids solution
BSA: Bovine serum albumin
CASP3: Caspase 3
CASP8: Caspase 8
CAT: Catalase
COCs: Cumulus-oocyte complexes
CTSB: Cathepsin B
CTSK: Cathepsin K
CTSS: Cathepsin S
CTSZ: Cathepsin Z
DAPI: 4′,6-diamidino-2-phenylindole
DCHFDA: 2′,7′-dichlorodihydrofluorescein diacetate
DNA: Deoxyribonucleic acid
EREG: Epiregulin
EtOH: Ethanol
FAS: Fas cell surface death receptor
FASLG: Fas ligand
FBS: Foetal bovine serum
FITC: Fluorescein isothiocyanate
GAPDH: Glyceraldehyde-3-phosphate dehydrogenase
GFPT1: Glutamine-fructose-6-phosphate transaminase 1
GFPT2: Glutamine-fructose-6-phosphate transaminase 2
GLUT1: Glucose transporter 1
GLUT4: Glucose transporter 4
GPX4: Glutathione peroxidase 4
GSH: Glutathione
HAS2: Hyaluronan synthase 2
hCG: Human chorionic gonadotropin
IETS: International Embryo Transfer Society
IVC: In vitro culture
IVF: In vitro fertilization
IVM: In vitro maturation
JC-1: Tetraethylbenzimidazolylcarbocyanine iodide dye
LDHA: Lactate dehydrogenase A
LH: Luteinizing hormone
MEM: Minimum essential medium non-essential amino acids solution
NAD: Nicotinamide adenine dinucleotide
PBS: Phosphate-buffered saline
PFKP: Phosphofructokinase
PGE_2_: Prostaglandin E_2_
PMSG: Pregnant mare’s serum gonadotropin
PTGER1: Prostaglandin E_2_ receptor 1
PTGER2: Prostaglandin E_2_ receptor 2
PTGER3: Prostaglandin E_2_ receptor 3
PTGER4: Prostaglandin E_2_ receptor 4
PTGES: Microsomal prostaglandin E synthase 1
PTGES2: Microsomal prostaglandin E synthase 2
PTGES3: Cytosolic prostaglandin E synthase
PTGS2: Prostaglandin endoperoxide synthase 2
PVA: Polyvinyl alcohol
real-time PCR: real-time Polymerase Chain Reaction
ROS: Reactive oxygen species
RT: Reverse transcription
RT: Room temperature
TNFAIP6: TNF alpha induced protein 6
TNFR1: Tumor necrosis factor receptor 1
TNFR2: Tumor necrosis factor receptor 2
TNFα: Tumor necrosis factor alpha
TUNEL: Terminal-uridine nick-end labeling
